# Real-world perioperative systemic treatment patterns and postoperative mortality in patients with upper tract urothelial carcinoma undergoing radical nephroureterectomy in Japan: WAKKA-UTUC study

**DOI:** 10.1007/s10147-026-03198-6

**Published:** 2026-09-19

**Authors:** Junichi Inokuchi, Jumpei Tokumaru, Daisuke Kawai, Ryotaro Ide, Hiroshi Komazaki, Hiroshi Kitagawa, Eiji Kikuchi

**Affiliations:** 1 Department of Urology, Graduate School of Medicine, University of the Ryukyus, 1076 Kiyuna, Ginowan, Okinawa 901-2720 Japan; 2 Medical Department, AstraZeneca K.K., Tokyo, Japan; 3 Department of Urology, St. Marianna University School of Medicine, Kanagawa, Japan

**Keywords:** Administrative claims database, Immunotherapy, Nephroureterectomy, Perioperative chemotherapy, Real-world evidence, Upper tract urothelial carcinoma

## Abstract

**Background:**

To characterize the longitudinal changes in perioperative systemic treatment patterns and postoperative mortality among Japanese patients with stage II–IV upper tract urothelial carcinoma (UTUC) who underwent radical nephroureterectomy (RNU).

**Methods:**

This retrospective observational study used a nationwide Japanese database to evaluate neoadjuvant chemotherapy (NAC) and adjuvant therapy (AT) before and after RNU. Treatment patterns, specific regimens, and 30- and 90-day postoperative mortality were analyzed descriptively across three periods: 2013–2017, 2018–2021, and 2022–2024.

**Results:**

Among 6,165 patients included in the study, the use of AT without prior NAC increased from 25.7% in 2013–2017 to 30.0% in 2022–2024, and the proportion of patients who received any NAC increased from 9.8% to 17.1%, respectively. By 2022–2024, gemcitabine plus carboplatin emerged as the most widely used regimen for NAC and AT (6.5% and 15.2% of the overall study population during this period [n = 1,482], respectively). Adjuvant nivolumab was administered to 142 patients (9.6% of all patients undergoing RNU in 2022–2024), but most (n = 84) had not received prior NAC. Postoperative mortality remained stable across the study periods (30-day mortality: 0.0%–0.5%; 90-day mortality: 0.3%–2.0%), with no increase in mortality among patients who received NAC.

**Conclusions:**

The use of perioperative systemic therapy for UTUC in Japan increased over the study period, with growing adoption of NAC, AT, and adjuvant nivolumab. Postoperative mortality remained stable, showing no notable association with NAC.

**Supplementary Information:**

The online version contains supplementary material available at https://doi.org/10.1007/s10147-026-03198-6.

## Introduction

Upper tract urothelial carcinoma (UTUC) accounts for approximately 5%‒10% of all urothelial carcinomas, with nearly 60% present as invasive disease with poor prognosis [1, 2]. The standard treatment for non-metastatic high-risk UTUC is radical nephroureterectomy (RNU), and in recent years, laparoscopic and robot-assisted approaches have largely replaced open surgery [3]. Despite definitive local treatment, patients treated with RNU alone remain at considerable risk of disease recurrence and death, supporting the rationale for effective perioperative chemotherapy [4, 5].

To improve survival outcomes, the integration of perioperative systemic therapy has become a major focus. Recent international and Japanese guidelines recommend adjuvant therapy (AT), prioritizing platinum-based chemotherapy and conditionally recommending nivolumab [2, 6]. In addition, neoadjuvant chemotherapy (NAC) has increasingly been incorporated into clinical practice [6–8]. The phase III POUT trial demonstrated significantly improved outcomes with platinum-based AT [9, 10], while the CheckMate 274 trial demonstrated improved disease-free survival (DFS) with adjuvant nivolumab in the overall trial population, of which patients with UTUC comprised only approximately 21% [11]. Notably, subgroup analyses of the CheckMate 274 trial did not demonstrate a clear DFS benefit for adjuvant nivolumab in patients whose primary tumor originated in the renal pelvis or ureter [12].

Delivering optimal perioperative chemotherapy in UTUC presents unique clinical challenges compared with bladder cancer. Patients with UTUC are generally older and frequently present with renal impairment. Furthermore, the loss of a renal unit during RNU causes a profound decline in renal function, often rendering patients ineligible for standard cisplatin-based regimens [13].

 Minimally invasive surgical techniques and immune checkpoint inhibitors have significantly changed the UTUC treatment landscape [2]. Clinical outcomes in this setting are influenced by multiple factors, such as the rates of perioperative NAC and AT administration, which are constrained by the decline in renal function following RNU, as well as specific regimen selection, dose adjustments, and postoperative mortality [8, 10, 13]. Despite these evolving paradigms, no reports have comprehensively examined perioperative treatment patterns and postoperative mortality specifically in patients with high-risk UTUC on a large scale in Japan. Therefore, this study sought to clarify the actual status and longitudinal transitions of perioperative treatment and postoperative mortality in a nationwide cohort exceeding 6,000 patients with advanced (stage II–IV) disease, in response to key changes in the clinical environment, specifically the widespread introduction of robot-assisted surgery, the reporting of the positive results of the POUT study in 2018 [9], and the approval of postoperative nivolumab in 2022.

## Patients and methods

### Study design

This retrospective observational study was conducted using the Medical Data Vision (MDV) database, which aggregates Diagnosis Procedure Combination (DPC) data, health claims, and administrative records from approximately 500 acute care hospitals in Japan (as of August 2022) [14]. Data were extracted for the period from January 1, 2012, to October 31, 2024 (Supplementary Fig. 1). The date of the patient’s RNU was defined as the index date (Day 0). Patients were followed through to October 31, 2024, or their last available record. Baseline patient characteristics, tumor staging, and comorbidities were evaluated during the preoperative look-back period (Day − 276 to Day − 1).

This study was conducted in accordance with the Declaration of Helsinki, the Ethical Guidelines for Medical and Biological Research Involving Human Subjects, and Good Pharmacoepidemiology Practices. The study protocol was reviewed and approved by the independent non-profit MINS Institutional Review Board (MINS-REC-250203; approved January 9, 2025). The analysis used de-identified data extracted from a commercially available administrative claims database; therefore, informed consent was not required under the Ethical Guidelines.

### Study population

Patients were included if they had a confirmed diagnosis of malignant neoplasm of renal pelvis (International Classification of Diseases, 10th Revision [ICD-10] code C65) or malignant neoplasm of ureter (ICD-10 code C66) prior to the index date, underwent RNU between January 1, 2013, and April 30, 2024, and were aged 18 years or older on the index date. In addition, patients were required to have documented tumor‒node‒metastasis (TNM) staging indicating stage II, III, or IV disease (without distant metastasis, i.e., M0) during the baseline period. Patients participating in clinical trials or with Mx or M1 disease were excluded.

### Data collection

The MDV database records demographic information, ICD-10 diagnosis codes, Japanese procedure codes, and prescriptions based on claims data and Anatomical Therapeutic Chemical classifications. Baseline characteristics, hospital size, and TNM stage (6th/7th [15, 16] or 8th [17] editions) were extracted from the look-back period. Because the MDV database cannot reliably differentiate clinical and pathological staging, stage classification may include both. Data regarding the type of surgical procedure (open vs. laparoscopic or robot-assisted RNU), specific NAC and AT regimens, treatment sequence, and postoperative vital status were collected from the corresponding treatment windows.

### Endpoints

The primary endpoint was the proportion of patients receiving each perioperative treatment pattern (NAC + RNU + AT, NAC + RNU, RNU + AT, or RNU alone). These patterns were evaluated across three distinct calendar periods defined by clinical milestones: 2013–2017 (pre-POUT trial era), 2018–2021 (post-POUT trial era), and 2022–2024 (post-nivolumab approval era).

Secondary endpoints included the distribution of specific NAC and AT regimens (or the absence of therapy) by study period, as well as the treatment sequences from NAC to AT observed during the most recent period. Systemic therapies were categorized into the following regimens: standard-dose gemcitabine + cisplatin (GC), reduced-dose GC, split-dose GC, gemcitabine + carboplatin (G-carboplatin), methotrexate + vinblastine + doxorubicin + cisplatin (MVAC), dose-dense MVAC (dd-MVAC), adjuvant nivolumab, or other regimens. Additional endpoints included 30- and 90-day post-RNU mortality.

### Statistical analysis

All analyses were conducted in the full analysis set (FAS), defined as all eligible patients who underwent RNU during the inclusion window. All statistical analyses were descriptive, and no formal hypothesis testing was performed. Categorical variables were summarized as counts and percentages, while continuous variables were presented as medians and interquartile ranges or minimum and maximum values. Mortality was calculated for the 30- and 90-day post-RNU periods. Missing data were not imputed. Analyses were performed using SAS Viya, version 3.5 (SAS Institute Inc., Cary, North Carolina, USA) and R, version 4.4.1 (R Foundation for Statistical Computing, Vienna, Austria).

## Results

### Patient disposition and characteristics

**Fig. 1:**
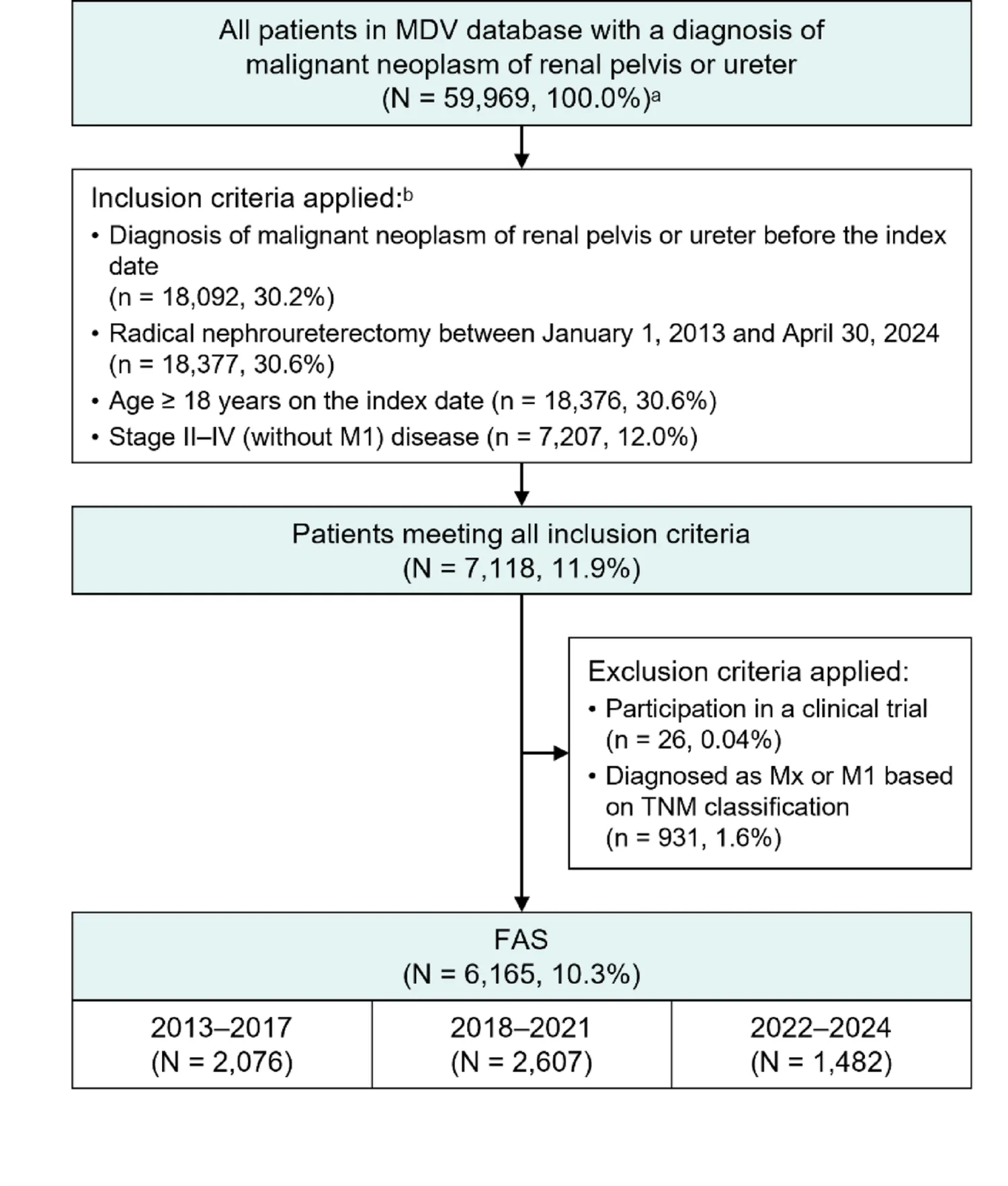
Patient disposition. ^a^The denominator is the number of patients in “All patients.” ^b^Patients may be counted in multiple inclusion criteria. *FAS* full analysis set, *MDV* Medical Data Vision, *TNM* tumor‒node‒metastasis

The FAS consisted of 6,165 patients who were stratified into three time periods: 2,076 in 2013–2017, 2,607 in 2018–2021, and 1,482 in 2022–2024 (Fig. 1). The median age of patients was 74.0 years in 2013–2017, 75.0 years in 2018–2021, and 76.0 years in 2022–2024 (Table 1). The proportion of males remained relatively stable at 66.8% in 2013–2017, 66.9% in 2018–2021, and 67.0% in 2022–2024. The primary tumor site was the renal pelvis in 47.9%–49.5% and the ureter in 54.1%–55.8% of patients (some had both). According to TNM classification, the proportion of patients with stage II disease was 36.9% in 2013–2017, 38.4% in 2018–2021, and 38.7% in 2022–2024. The proportions of patients with stage III disease were 45.8%, 46.7%, and 46.6%, respectively, and the proportions of patients with stage IV disease were 17.3%, 14.9%, and 14.8%, respectively. The proportion of patients who underwent laparoscopic or robot-assisted surgery increased substantially from 70.8% in 2013–2017 to 91.6% in 2022–2024 (Table 1).

Across all study periods, patients undergoing RNU alone tended to be older than those receiving perioperative chemotherapy; for example, in 2022–2024, the median age of patients receiving NAC + RNU + AT was 74 years, whereas patients undergoing RNU alone had a higher median age of 79 years (Supplementary Table 1).

**Table 1:** Baseline patient characteristics by study period

Characteristic	2013–2017 (N = 2,076)	2018–2021 (N = 2,607)	2022–2024 (N = 1,482)
Age, median (min, max), years	74.0 (33, 94)	75.0 (27, 97)	76.0 (39, 96)
Male	1,386 (66.8)	1,743 (66.9)	993 (67.0)
*Surgery type*			
Open RNU	607 (29.2)	480 (18.4)	125 (8.4)
LRN/RANU	1,469 (70.8)	2,127 (81.6)	1,357 (91.6)
*Primary site of tumor* ^a^			
Renal pelvis	995 (47.9)	1,291 (49.5)	712 (48.0)
Ureter	1,146 (55.2)	1,410 (54.1)	827 (55.8)
Smoking history	851 (41.0)	1,130 (43.3)	670 (45.2)
*TNM stage*			
Stage II	766 (36.9)	1,001 (38.4)	573 (38.7)
Stage III	951 (45.8)	1,217 (46.7)	690 (46.6)
Stage IV	359 (17.3)	389 (14.9)	219 (14.8)
*Hospital size*			
< 200 beds	87 (4.2)	71 (2.7)	45 (3.0)
200**–**499 beds	1,071 (51.6)	1,388 (53.2)	709 (47.8)
≥ 500 beds	918 (44.2)	1,148 (44.0)	728 (49.1)
*Time to surgery from start date of final NAC, days*			
n	192	345	244
Median (min, max)	45.0 (13, 168)	43.0 (19, 127)	44.0 (13, 142)
*Time to start of AT from surgery, days*			
n	540	739	506
Median (min, max)	43.0 (8, 124)	43.0 (12, 124)	47.0 (18, 124)

Data are presented as n (%) unless specified otherwise

TNM stage classification was assigned according to the UICC TNM staging system, using the 6th/7th editions [15, 16] or the 8th edition [17]

*AT* adjuvant therapy, *LRN* laparoscopic radical nephroureterectomy, *NAC* neoadjuvant chemotherapy, *RANU* robot-assisted radical nephroureterectomy, *RNU* radical nephroureterectomy, *TNM* tumor‒node‒metastasis, *UICC* Union for International Cancer Control

^a^Patients with codes for both tumor sites were included in both categories

**Table 2:** 30- and 90-day postoperative mortality by use of NAC and study period

Treatment pattern	2013–2017	2018–2021	2022–2024
*30*-*day mortality*			
NAC	1/205 (0.5)	1/363 (0.3)	0/254 (0.0)
No NAC	2/1,871 (0.1)	6/2,244 (0.3)	1/1,228 (0.1)
*90*-*day mortality*			
NAC	4/205 (2.0)	1/363 (0.3)	1/254 (0.4)
No NAC	19/1,871 (1.0)	20/2,244 (0.9)	9/1,228 (0.7)

Data are presented as n/N (%)

*NAC* neoadjuvant chemotherapy

### Perioperative treatment patterns over time and by disease stage

**Fig. 2:**
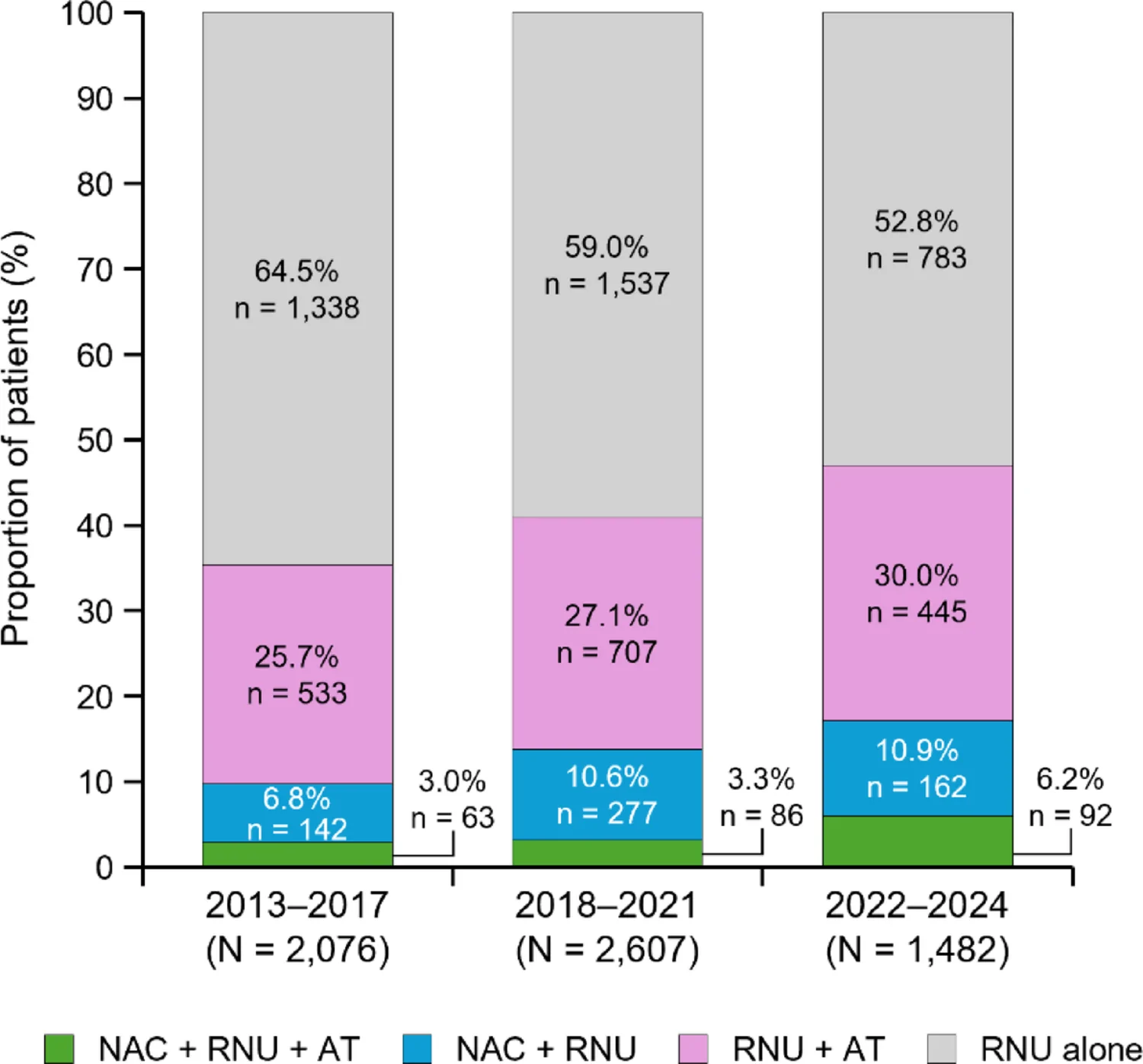
Perioperative treatment patterns over time (2013–2024). *AT* adjuvant therapy, *NAC* neoadjuvant chemotherapy, *RNU* radical nephroureterectomy

The proportion of patients receiving RNU + AT (without NAC) increased from 25.7% in 2013–2017 to 27.1% in 2018–2021, and to 30.0% in 2022–2024 (Fig. 2). Any NAC use (i.e., NAC + RNU + AT or NAC + RNU without AT) increased from 9.8% to 13.9%, and to 17.1%, respectively. While RNU alone remained the most frequently used treatment approach overall, its use declined across the three study periods.

**Fig. 3:**
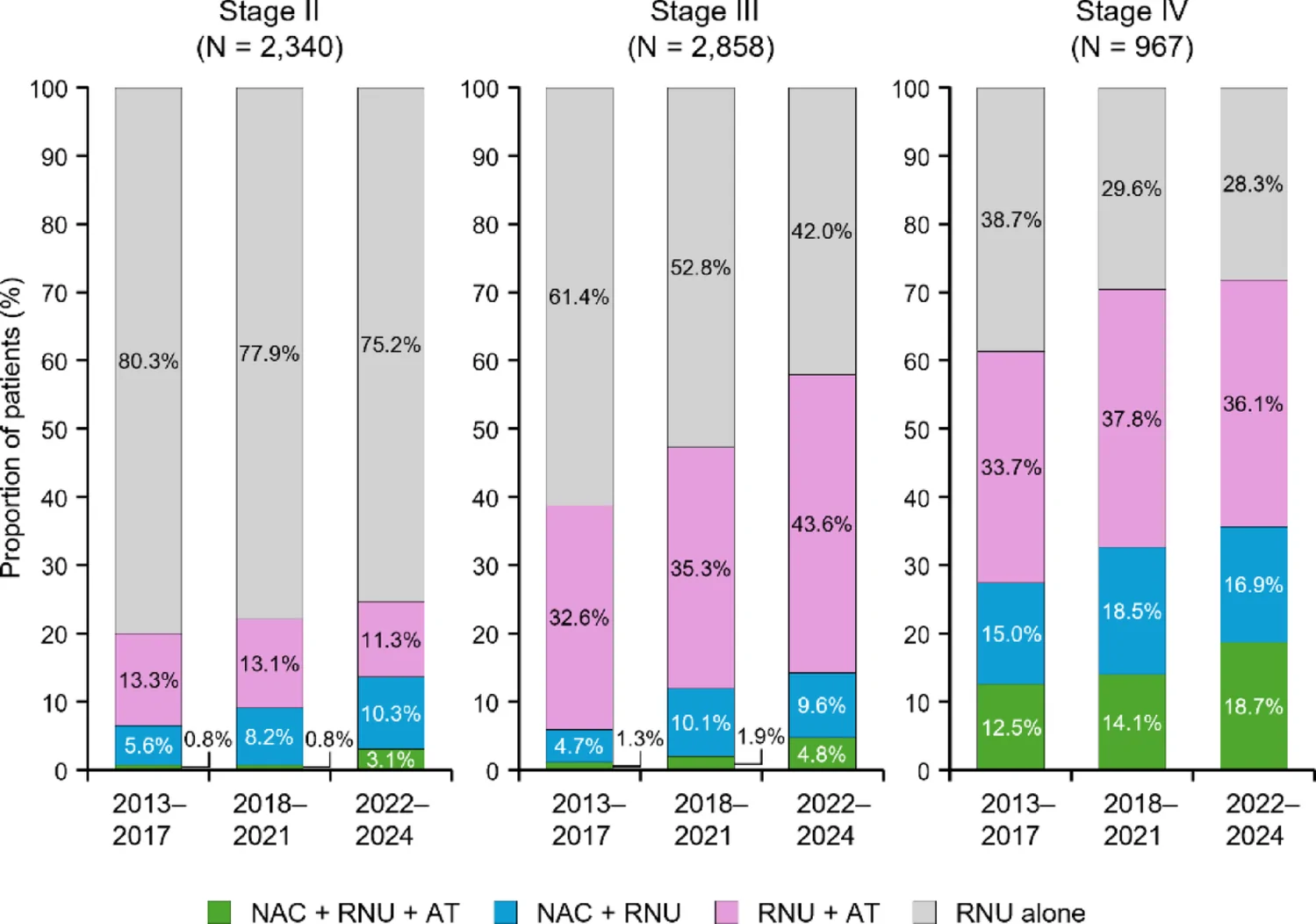
Perioperative treatment patterns by disease stage (2013–2024). TNM stage classification was assigned according to the UICC TNM staging system, using the 6th/7th editions [15, 16] or the 8th edition [17]. *AT* adjuvant therapy, *NAC* neoadjuvant chemotherapy, *RNU* radical nephroureterectomy, *TNM* tumor‒node‒metastasis, *UICC* Union for International Cancer Control

Trends in treatment patterns demonstrated some variation when patients were stratified by disease stage (Fig. 3). In stage II disease, any NAC use was 6.4% in 2013–2017, 9.0% in 2018–2021, and 13.4% in 2022–2024. RNU + AT use was 13.3%, 13.1%, and 11.3%, respectively. In stage III disease, any NAC use increased across periods (6.0%, 12.0%, and 14.4%), as did RNU + AT (32.6%, 35.3%, and 43.6%). In stage IV disease (M0), any NAC use increased across periods (27.5%, 32.6%, and 35.6%), while RNU + AT use was 33.7%, 37.8%, and 36.1%.

### Neoadjuvant and adjuvant systemic therapy regimens

**Fig. 4:**
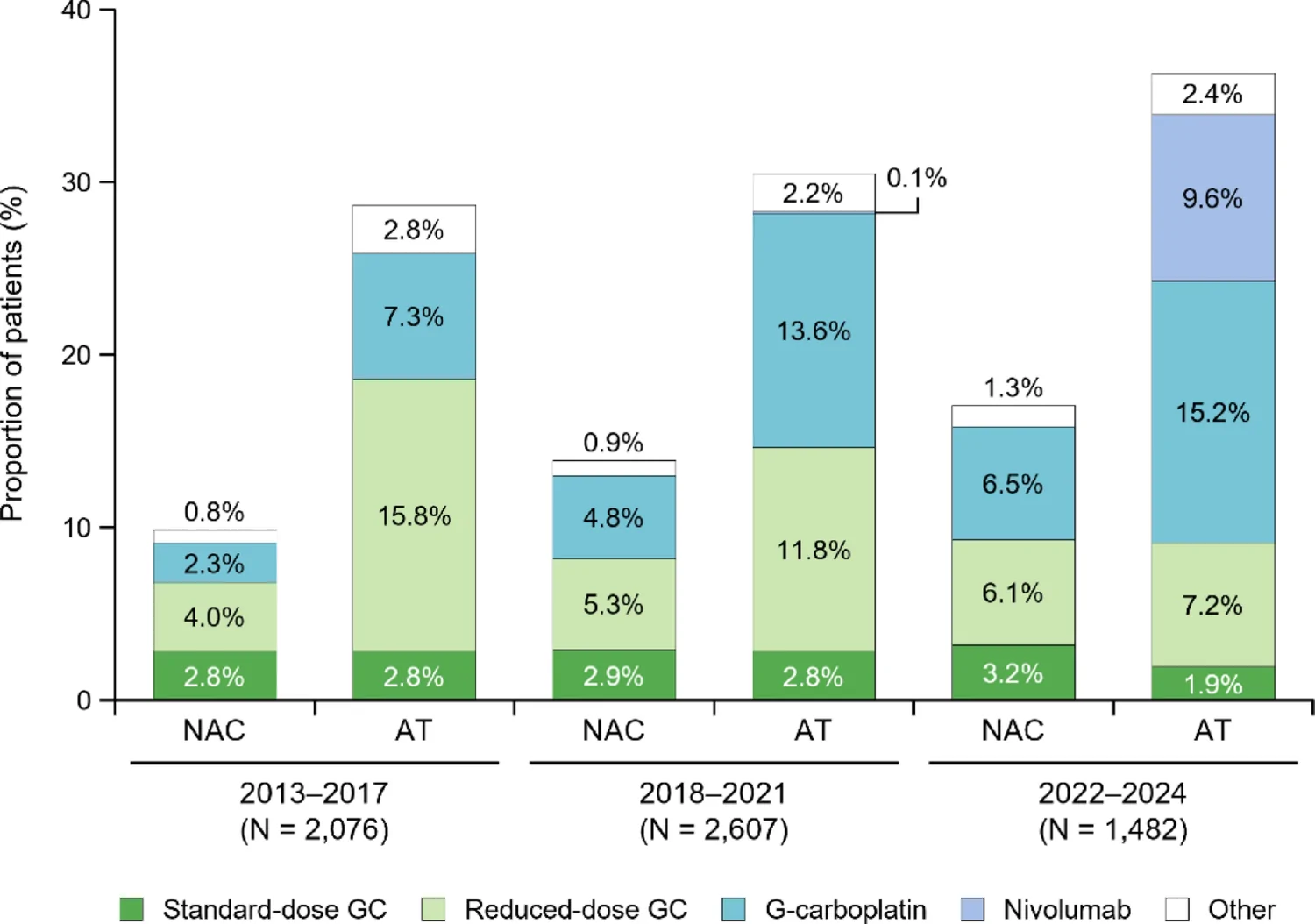
Proportions of patients receiving specific neoadjuvant and adjuvant therapy regimens (2013–2024). The “Other” category includes split-dose GC, dd-MVAC, MVAC, and other regimens not shown individually. *AT* adjuvant therapy, *dd-MVAC* dose-dense methotrexate + vinblastine + doxorubicin + cisplatin, *G-carboplatin* gemcitabine + carboplatin, *GC* gemcitabine + cisplatin, *MVAC* methotrexate + vinblastine + doxorubicin + cisplatin, *NAC* neoadjuvant chemotherapy

Overall, the use of specific regimens, such as standard-dose GC, reduced-dose GC, and G-carboplatin, demonstrated dynamic shifts over the three study periods (Fig. 4). In the neoadjuvant setting, although the proportions of patients receiving these specific NAC regimens were low, their use increased over time (Supplementary Table 2). In the adjuvant setting, the use of reduced-dose GC decreased from 15.8% in 2013–2017 to 7.2% in 2022–2024, while the proportion of patients receiving standard-dose GC remained persistently low (Supplementary Table 3). Ultimately, G-carboplatin emerged as the most widely used regimen for both NAC (6.5%) and AT (15.2%) in 2022–2024.

**Fig. 5:**
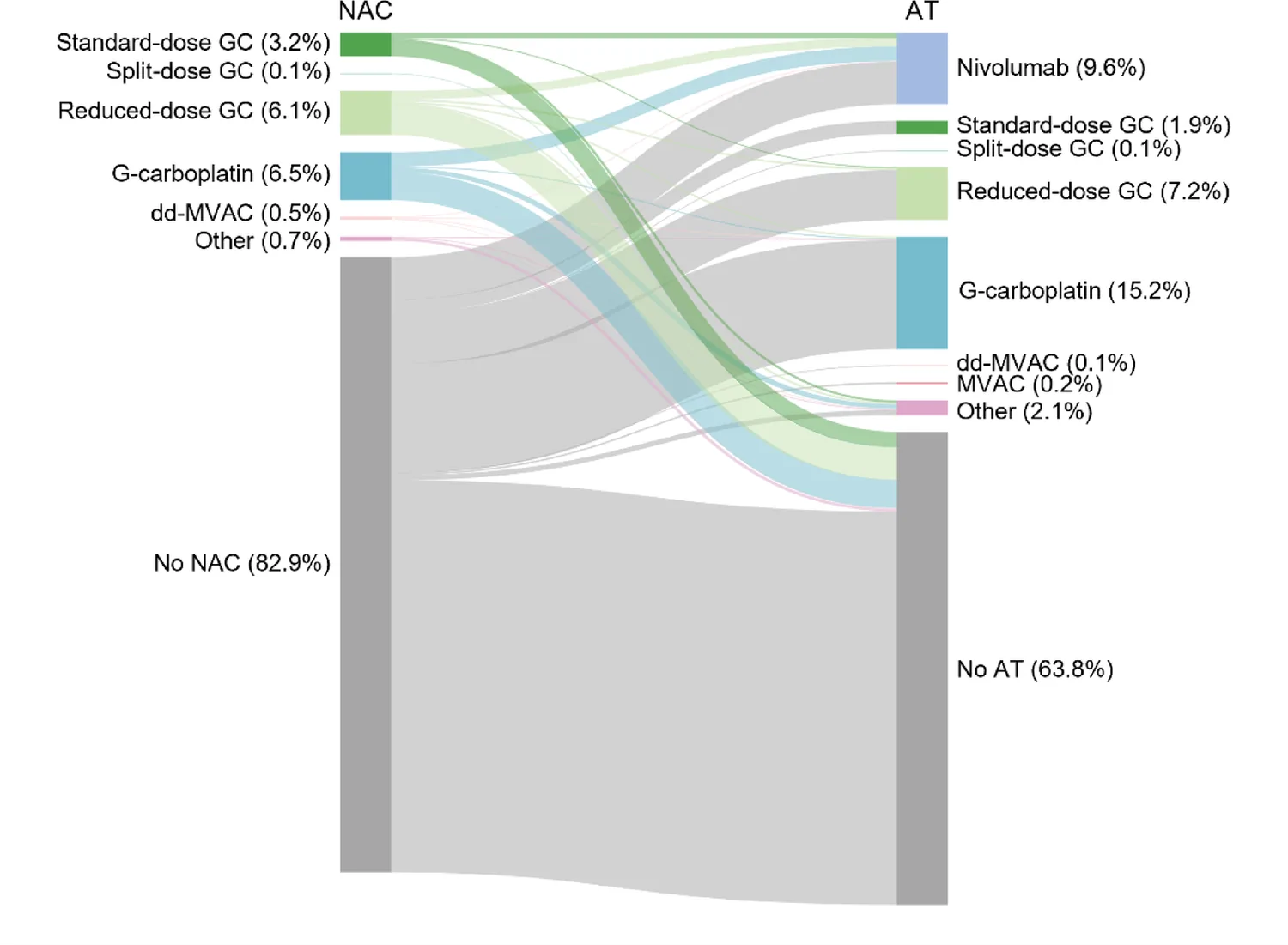
Treatment sequence from neoadjuvant chemotherapy to adjuvant therapy (2022–2024). *AT* adjuvant therapy, *dd-MVAC* dose-dense methotrexate + vinblastine + doxorubicin + cisplatin, *G-carboplatin* gemcitabine + carboplatin, *GC* gemcitabine + cisplatin, *MVAC* methotrexate + vinblastine + doxorubicin + cisplatin, *NAC* neoadjuvant chemotherapy

Figure 5 shows the treatment flow patterns for 2022–2024. The majority of AT regimens in patients without NAC were chemotherapies, including G-carboplatin and reduced-dose GC, while nivolumab was used in only 9.6% (n = 142) of patients. Among these 142 patients, 58 received prior NAC, while 84 received no NAC.

### Postoperative mortality

Postoperative mortality demonstrated no notable changes between the study periods (Table 2). Specifically, the 30-day mortality ranged from 0.1% to 0.3% and the 90-day mortality ranged from 0.7% to 1.0% in the no-NAC group. There was no numerical increase in mortality among patients who received NAC.

## Discussion

Given UTUC’s rarity [2], enrolling enough patients for robust clinical research at individual institutions is difficult, making large-scale database studies essential for understanding real-world outcomes of UTUC [18]. In Japan, the MDV database serves as a highly representative data source for this purpose, capturing data from approximately 35% of all DPC hospitals nationwide. This study represents the largest descriptive analysis conducted in Japan to evaluate perioperative systemic therapy and postoperative mortality, capturing over 6,000 patients with UTUC treated with RNU between 2013 and 2024. Our findings demonstrate that the use of perioperative systemic therapy has gradually increased over the study period, reflecting a growing integration of both NAC and AT into clinical practice. Furthermore, postoperative mortality remained stable during this period, with no numerical increase observed among patients who received NAC.

Our data indicate that higher disease stages were associated with greater proportions of patients receiving systemic therapy and higher treatment intensity. However, RNU alone continued to be common across all stages. In 2022–2024, a substantial proportion of patients with stage II UTUC underwent RNU alone. Although many patients with stage III or IV disease received RNU alone, possibly due to poor preoperative renal function, advanced age, or other baseline characteristics that increase the risk of postoperative complications [19–21], the use of AT progressively increased over time. This trend in AT use was likely influenced by the POUT and CheckMate 274 trials [10, 11]. Although the proportion of patients who received NAC remained smaller than that of AT, the use of NAC increased. During most of the study period, evidence supporting NAC in UTUC was limited, and its role had not been firmly established in the Japanese Urological Association (JUA) guidelines available at the time [6]. Furthermore, lacking large-scale UTUC*-*specific randomized controlled trials, clinical practice largely extrapolated from bladder cancer. In bladder cancer, there was evidence supporting perioperative cisplatin-based chemotherapy, particularly in the neoadjuvant setting and, to a lesser extent, in the adjuvant setting; by contrast, the benefit of carboplatin-based regimens had not been established. As a result, a substantial proportion of cisplatin-ineligible patients were likely treated with surgery alone without perioperative systemic therapy. However, the utility of NAC as a preoperative treatment was increasingly recognized, given that renal function often declines after RNU [13, 22], and because of the influence of evidence from bladder cancer [23]. In addition, the increase in the proportion of patients who received NAC + AT was also likely influenced by the CheckMate 274 trial [11]. Further evidence on the use of NAC in UTUC is needed, and it will be important to evaluate ongoing changes in treatment practices following the updated Japanese guidelines’ incorporation of new evidence [24].

Among specific perioperative chemotherapy regimens, platinum-based chemotherapy was commonly used as NAC and AT. The proportion of patients who received standard-dose GC remained low, while reduced-dose GC and G-carboplatin were more common. This limited use of standard-dose GC, particularly as AT, is largely attributable to the decreased renal function resulting from unilateral nephrectomy. Between 2013–2017 and 2022–2024, the use of G-carboplatin increased approximately twofold as AT. The trend toward increasing use of G-carboplatin in AT is closely related to the treatment framework established following the reporting of the positive results of the POUT trial in 2018 [9, 10]. In that trial, gemcitabine and cisplatin were permitted for patients with a postoperative glomerular filtration rate (GFR) of ≥ 50 mL/min, whereas carboplatin was substituted in patients with a GFR of 30‒49 mL/min. Consequently, G-carboplatin became a viable option for patients who were unfit for cisplatin. Although the fundamental AT regimen remains GC and the POUT trial lacked the statistical power to formally evaluate the independent efficacy of G-carboplatin, the trial presented a framework for selecting adjuvant therapy even under postoperative renal constraints. This POUT trial framework likely contributed to the increased proportion of G-carboplatin use as an AT observed in real-world clinical practice. At the same time, G-carboplatin was the most common neoadjuvant regimen (6.5%) in the most recent period (2022–2024). The increasing use of carboplatin in the NAC setting in real-world clinical practice, despite the extremely limited evidence supporting it, highlights a significant unmet need for further evidence to guide neoadjuvant treatment selection.

In 2022–2024, most AT regimens for patients without NAC consisted of conventional chemotherapy, while nivolumab use remained limited. This likely reflects domestic JUA guidelines, which recommend systemic chemotherapy as the primary option for AT in patients without NAC, with a weaker recommendation for nivolumab [6]. Furthermore, international urological guidelines recommend adjuvant nivolumab primarily for patients who have already received platinum-based NAC, as well as for high-risk patients who are ineligible for or refuse perioperative cisplatin [2]. This sequenced approach is largely supported by subgroup analyses of the CheckMate 274 trial, which did not demonstrate a clear DFS benefit for adjuvant nivolumab in patients without prior NAC or in the subgroup of patients with cancer of the renal pelvis or ureter [11]. Consequently, the Optimal Use Promotion Guideline recommends its use preferentially following NAC [25]. However, approximately 60% of the patients who received adjuvant nivolumab had not received prior NAC. The reasons for this treatment pattern could not be determined from the MDV database because information regarding renal function, treatment eligibility, and physician decision-making was unavailable. Potential explanations may include cisplatin ineligibility, advanced age, comorbidities, patient preference, or evolving treatment practices. Further studies are warranted to better understand treatment selection in this population.

To the best of our knowledge, this is the largest report to describe postoperative mortality in UTUC using nationwide data in Japan. Previous population-level studies in North America have reported a 30-day mortality of 1.8% [26] and a 90-day mortality of 4.4% [27] following RNU. In the present study, the 30-day and 90-day mortality, regardless of whether the patients received NAC, was consistently below 0.5% and 2.0%, respectively, across all three time periods. The lower mortality observed in our cohort compared with the earlier studies is likely attributable to the widespread adoption of less invasive techniques, such as laparoscopic and robot-assisted surgery, as well as to general advancements in perioperative care. Furthermore, postoperative mortality did not show a numerical increase, despite increased use of NAC during the study period. This likely reflects appropriate patient selection and perioperative management.

This study has several limitations. First, owing to the nature of the claims database, detailed clinical information, such as renal function and performance status, was not available. In addition, inherent limitations related to diagnostic coding and operational definitions mean that certain non-UTUC malignancies (e.g., collecting duct carcinoma) may have been inadvertently included in the cohort. Furthermore, the MDV database did not allow consistent differentiation between clinical and pathological staging. Therefore, some degree of stage misclassification may have occurred, which should be considered when interpreting stage-specific findings. Second, the MDV database tracks patient data only within the same medical institution, and the number of participating institutions may change over time. Consequently, there is a potential for underestimating postoperative outcomes, including 90-day mortality, or misclassifying treatment patterns if patients are transferred to a different hospital following surgery. However, because UTUC is typically managed continuously at a single institution, the prevalence of such underestimation is likely to be small. Third, the generalizability of our findings may be limited. Because the MDV database is primarily sourced from DPC hospitals, the study cohort may not perfectly represent the entire Japanese UTUC population. However, because complex perioperative UTUC management predominantly occurs at large acute care facilities (mostly DPC hospitals) equipped to handle sudden clinical changes, these findings likely reflect the current real-world status of UTUC treatment in Japan. Fourth, systemic treatments were classified based solely on the initial treatment regimen, which may not capture subsequent regimen modifications. Finally, we evaluated treatment patterns across predefined multi-year periods to ensure sufficient sample sizes around major paradigm shifts. Consequently, it is difficult to fully differentiate the direct impact of new clinical evidence from gradual secular trends.

## Conclusions

The longitudinal evaluation of a large-scale nationwide database revealed that the use of both AT and NAC for UTUC gradually increased over the study period, and postoperative mortality did not show a numerical increase despite increased use of NAC. These findings reflect evolving real-world clinical practice, where treatment selection is personalized to balance oncological benefit and patient-specific factors. Further evidence regarding new pharmacological treatments for UTUC is highly anticipated to guide these treatment decisions.

## Supplementary Information

Below is the link to the electronic supplementary material.

Supplementary Material 1

## Data Availability

The data used in this study cannot be shared with external researchers due to the terms of the research contract with Medical Data Vision. Researchers may contact Medical Data Vision directly for all data requests (https://en.mdv.co.jp/).
