# Supplementary material for: Real-world perioperative systemic treatment patterns and postoperative mortality in patients with upper tract urothelial carcinoma undergoing radical nephroureterectomy in Japan: WAKKA-UTUC study
Source: Int J Clin Oncol. 2026 Sep 19;31(11):2823–32. doi: 10.1007/s10147-026-03198-6 (PMC13710618; doi:10.1007/s10147-026-03198-6)
Supplement: Supplementary file 1 [file 10147_2026_3198_MOESM1_ESM.docx]

# Supplementary materials

**Real-world perioperative systemic treatment patterns and postoperative mortality in patients with upper tract urothelial carcinoma undergoing radical nephroureterectomy in Japan: WAKKA-UTUC study**

**Authors:** Junichi Inokuchi, Jumpei Tokumaru, Daisuke Kawai, Ryotaro Ide, Hiroshi Komazaki, Hiroshi Kitagawa, Eiji Kikuchi

**Corresponding author:** Junichi Inokuchi

**Email address:** [junino@cs.u-ryukyu.ac.jp](mailto:junino@cs.u-ryukyu.ac.jp)

| **Contents** | **Page** |
| --- | --- |
| **Supplementary Fig. 1** Study design | 2 |
| **Supplementary Table 1** Baseline patient characteristics by study period and treatment pattern | 3 |
| **Supplementary Table 2** Proportions of patients receiving each neoadjuvant therapy regimen | 5 |
| **Supplementary Table 3** Proportions of patients receiving each adjuvant therapy regimen | 6 |
| **References** | 7 |

## Supplementary Fig. 1 Study design.

Days were counted relative to the index date (Day 0), corresponding to the date of the first radical nephroureterectomy

*AT* adjuvant therapy, *ICD-10* International Classification of Diseases, 10th Revision, *NAC* neoadjuvant chemotherapy, *RNU* radical nephroureterectomy, *TNM* tumor‒node‒metastasis

## Supplementary Table 1 Baseline patient characteristics by study period and treatment pattern

| **Characteristic** | **2013–2017** | | | | **2018–2021** | | | | **2022–2024** | | | |
| --- | --- | --- | --- | --- | --- | --- | --- | --- | --- | --- | --- | --- |
|  | **NAC + RNU + AT (n = 63)** | **NAC + RNU (n = 142)** | **RNU + AT (n = 533)** | **RNU alone (n = 1,338)** | **NAC + RNU + AT (n = 86)** | **NAC + RNU (n = 277)** | **RNU + AT (n = 707)** | **RNU alone (n = 1,537)** | **NAC + RNU + AT (n = 92)** | **NAC + RNU (n = 162)** | **RNU + AT (n = 445)** | **RNU alone (n = 783)** |
| Age, median (min, max), years | 72.0  (42, 88) | 71.5  (51, 88) | 70.0  (38, 89) | 77.0  (33, 94) | 70.0  (27, 89) | 73.0  (42, 89) | 72.0  (40, 92) | 77.0  (38, 97) | 74.0  (49, 86) | 74.0  (51, 89) | 74.0  (39, 89) | 79.0  (49, 96) |
| Male | 46 (73.0) | 88 (62.0) | 380 (71.3) | 872 (65.2) | 54 (62.8) | 192 (69.3) | 479 (67.8) | 1,018 (66.2) | 61 (66.3) | 113 (69.8) | 300 (67.4) | 519 (66.3) |
| Surgery type |  |  |  |  |  |  |  |  |  |  |  |  |
| Open RNU | 34 (54.0) | 67 (47.2) | 137 (25.7) | 369 (27.6) | 32 (37.2) | 65 (23.5) | 134 (19.0) | 249 (16.2) | 13 (14.1) | 15 (9.3) | 38 (8.5) | 59 (7.5) |
| LRN/RANU | 29 (46.0) | 75 (52.8) | 396 (74.3) | 969 (72.4) | 54 (62.8) | 212 (76.5) | 573 (81.0) | 1,288 (83.8) | 79 (85.9) | 147 (90.7) | 407 (91.5) | 724 (92.5) |
| Primary site of tumor^a^ |  |  |  |  |  |  |  |  |  |  |  |  |
| Renal pelvis | 35 (55.6) | 67 (47.2) | 236 (44.3) | 657 (49.1) | 38 (44.2) | 133 (48.0) | 337 (47.7) | 783 (50.9) | 44 (47.8) | 71 (43.8) | 214 (48.1) | 383 (48.9) |
| Ureter | 32 (50.8) | 84 (59.2) | 314 (58.9) | 716 (53.5) | 53 (61.6) | 152 (54.9) | 396 (56.0) | 809 (52.6) | 56 (60.9) | 99 (61.1) | 247 (55.5) | 425 (54.3) |
| Smoking history | 27 (42.9) | 54 (38.0) | 253 (47.5) | 517 (38.6) | 38 (44.2) | 125 (45.1) | 310 (43.8) | 657 (42.7) | 37 (40.2) | 77 (47.5) | 199 (44.7) | 357 (45.6) |
| TNM stage |  |  |  |  |  |  |  |  |  |  |  |  |
| Stage II | 6 (9.5) | 43 (30.3) | 102 (19.1) | 615 (46.0) | 8 (9.3) | 82 (29.6) | 131 (18.5) | 780 (50.7) | 18 (19.6) | 59 (36.4) | 65 (14.6) | 431 (55.0) |
| Stage III | 12 (19.0) | 45 (31.7) | 310 (58.2) | 584 (43.6) | 23 (26.7) | 123 (44.4) | 429 (60.7) | 642 (41.8) | 33 (35.9) | 66 (40.7) | 301 (67.6) | 290 (37.0) |
| Stage IV | 45 (71.4) | 54 (38.0) | 121 (22.7) | 139 (10.4) | 55 (64.0) | 72 (26.0) | 147 (20.8) | 115 (7.5) | 41 (44.6) | 37 (22.8) | 79 (17.8) | 62 (7.9) |
| Hospital size |  |  |  |  |  |  |  |  |  |  |  |  |
| <200 beds | 1 (1.6) | 5 (3.5) | 24 (4.5) | 57 (4.3) | 3 (3.5) | 8 (2.9) | 15 (2.1) | 45 (2.9) | 0 (0.0) | 5 (3.1) | 10 (2.2) | 30 (3.8) |
| 200–499 beds | 28 (44.4) | 62 (43.7) | 288 (54.0) | 693 (51.8) | 45 (52.3) | 115 (41.5) | 379 (53.6) | 849 (55.2) | 33 (35.9) | 62 (38.3) | 207 (46.5) | 407 (52.0) |
| ≥500 beds | 34 (54.0) | 75 (52.8) | 221 (41.5) | 588 (43.9) | 38 (44.2) | 154 (55.6) | 313 (44.3) | 643 (41.8) | 59 (64.1) | 95 (58.6) | 228 (51.2) | 346 (44.2) |
| Time to surgery from start date of final NAC, days |  |  |  |  |  |  |  |  |  |  |  |  |
| Median (min, max) | 43.0  (13, 94) | 47.0  (22, 168) | – | – | 40.0  (20, 127) | 43.0  (19, 125) | – | – | 44.0  (13, 106) | 44.0  (17, 142) | – | – |
| Time to start of AT from surgery, days |  |  |  |  |  |  |  |  |  |  |  |  |
| Median (min, max) | 47.5  (10, 123) | – | 42.0  (8, 124) | – | 44.0  (21, 124) | – | 43.0  (12, 124) | – | 54.0  (23, 112) | – | 47.0  (18, 124) | – |

Data are presented as n (%), unless specified otherwise

TNM stage classification was assigned according to the UICC TNM staging system, using the 6th/7th editions [1,2] or the 8th edition [3]

^a^Patients with codes for both tumor sites were included in both categories

*AT* adjuvant therapy, *LRN* laparoscopic radical nephroureterectomy, *NAC* neoadjuvant chemotherapy, *RANU* robot-assisted radical nephroureterectomy, *RNU* radical nephroureterectomy, *TNM* tumor‒node‒metastasis, *UICC* Union for International Cancer Control

## Supplementary Table 2 Proportions of patients receiving each neoadjuvant therapy regimen

| **Regimen type** | **2013–2017 (N = 2,076)** | **2018–2021 (N = 2,607)** | **2022–2024 (N = 1,482)** |
| --- | --- | --- | --- |
| Standard-dose GC | 59 (2.8) | 76 (2.9) | 48 (3.2) |
| Split-dose GC | 3 (0.1) | 3 (0.1) | 2 (0.1) |
| Reduced-dose GC | 83 (4.0) | 139 (5.3) | 90 (6.1) |
| G-carboplatin | 47 (2.3) | 124 (4.8) | 97 (6.5) |
| dd-MVAC | 0 (0.0) | 3 (0.1) | 7 (0.5) |
| MVAC | 0 (0.0) | 0 (0.0) | 0 (0.0) |
| Other regimens | 13 (0.6) | 18 (0.7) | 10 (0.7) |
| No NAC | 1,871 (90.1) | 2,244 (86.1) | 1,228 (82.9) |

Data are presented as n (%)

*dd-MVAC* dose-dense methotrexate + vinblastine + doxorubicin + cisplatin, *G-carboplatin* gemcitabine + carboplatin, *GC* gemcitabine + cisplatin, *MVAC* methotrexate + vinblastine + doxorubicin + cisplatin, *NAC* neoadjuvant chemotherapy

## Supplementary Table 3 Proportions of patients receiving each adjuvant therapy regimen

| **Regimen type** | **2013–2017 (N = 2,076)** | **2018–2021 (N = 2,607)** | **2022–2024 (N = 1,482)** |
| --- | --- | --- | --- |
| Standard-dose GC | 59 (2.8) | 72 (2.8) | 28 (1.9) |
| Split-dose GC | 1 (0.0) | 2 (0.1) | 1 (0.1) |
| Reduced-dose GC | 327 (15.8) | 307 (11.8) | 106 (7.2) |
| G-carboplatin | 151 (7.3) | 354 (13.6) | 225 (15.2) |
| dd-MVAC | 0 (0.0) | 0 (0.0) | 1 (0.1) |
| MVAC | 2 (0.1) | 1 (0.0) | 3 (0.2) |
| Nivolumab | 0 (0.0) | 3 (0.1) | 142 (9.6) |
| Other regimens | 56 (2.7) | 54 (2.1) | 31 (2.1) |
| No AT | 1,480 (71.3) | 1,814 (69.6) | 945 (63.8) |

Data are presented as n (%)

*AT* adjuvant therapy, *dd-MVAC* dose-dense methotrexate + vinblastine + doxorubicin + cisplatin, *G-carboplatin* gemcitabine + carboplatin, *GC* gemcitabine + cisplatin, *MVAC* methotrexate + vinblastine + doxorubicin + cisplatin

# References

1. Sobin LH, Wittekind C, International Union Against Cancer (2002) TNM Classification of Malignant Tumours. vol 626, 6th edn. John Wiley & Sons, Ltd, Chichester, UK

2. Sobin LH, Gospodarowicz MK, Wittekind C (2009) TNM Classification of Malignant Tumours. 7th edn. John Wiley & Sons Ltd, Chichester, UK

3. Brierley JD, Gospodarowicz MK, Wittekind C (2017) TNM Classification of Malignant Tumours. 8th edn. John Wiley & Sons, Ltd, Chichester, UK
